# Fatty acid regio-specificity of triacylglycerol molecules may affect plasma lipid responses to dietary fats—a randomised controlled cross-over trial

**DOI:** 10.1038/s41430-019-0452-7

**Published:** 2019-06-21

**Authors:** Welma Stonehouse, Bianca Benassi-Evans, Genevieve James-Martin, Mahinda Abeywardena

**Affiliations:** Commonwealth Scientific Industrial Research Organisation, Health and Biosecurity, Adelaide, South Australia Australia

**Keywords:** Nutrition, Randomized controlled trials, Risk factors

## Abstract

**Background/Objectives:**

Hypercholesterolaemic effects of saturated fatty acids (SFA) may be influenced not only by the chain length, but also by their specific location within the triacylglycerol (TAG) molecule. We examined the hypothesis that dietary fats rich in SFA, but containing mostly unsaturated fatty acids in the *sn*-2 position with most SFA in *sn*-1 and -3 (palm olein [PO] and cocoa butter [CB]) will have similar serum lipid outcomes to unsaturated olive oil (OO).

**Subjects/Methods:**

Thirty-eight participants (20–40 yr, 18.5– ≤ 27.5 kg/m^2^) completed a 4-week randomised 3 × 3 crossover feeding intervention, preceded by 2-week run-in and separated by 2-week washout periods. Background diet contained 35 percentage of total energy (%E) fat, 18%E protein, 48%E carbohydrates, differing in test fats only (palm olein (PO), CB, OO; 20%E). Total cholesterol (TC)/high density lipoprotein cholesterol (HDL-C) ratio and related variables; TC, HDL-C, low density lipoprotein cholesterol (LDL-C), TAG, apoA1, ApoB, ApoA1 (apolipoprotein A1)/ApoB (apolipoprotein B), lipoprotein (a) (Lp(a)), NEFA, LDL sub-fractions, were assessed pre- and post-intervention. Data were analysed using mixed effects longitudinal models with a *P*-value < 0.05 considered significant.

**Results:**

Changes in plasma fatty acids (*P* < 0.05) confirmed compliance; C18:1 increased with OO compared to PO and CB; C16:0 decreased with OO and C18:0 increased following CB. No differences were seen for TC/HDL-C (mean [95%CI] change for PO, 0.08[0.00, 0.15] mmol/L; CB, 0.06 [−0.05, 0.16] mmol/L; and OO, −0.01 [−0.15, 0.13] mmol/L; *P* = 0.53] or any other parameter including LDL sub-fractions. OO decreased IDL-A compared to PO (−2.2 [−4.31, −0.21] mg/dL, *P* = 0.03).

**Conclusion:**

In healthy young participants, plasma lipid responses to PO and CB, enriched in SFA but having primarily unsaturated fatty acid in the *sn*-2 position of TAG, did not differ from OO.

## Introduction

Cardiovascular disease (CVD) remains the leading cause of mortality worldwide, accounting for one-third of all global deaths [1]. Reducing saturated fatty acids (SFA) is a key dietary strategy for lowering CVD risk, based primarily on its cholesterol-raising effects; [2] although for many countries the intake of SFA remain higher than recommended intakes [3].

SFA are used in the manufacturing of a number of foods and confectionary products, as they provide desirable and complementary attributes and functions, not equalled by unsaturated fats. For example, SFA are more oxidatively stable and contributes significantly more to the texture, flavour, sensory and mouth feel properties of food compared to unsaturated fats. Furthermore, most SFA can be transformed into margarines and shortenings without the need for hydrogenation. This is an important quality as these fats can replace hydrogenated fats, previously used for this purpose, but no longer recognised as safe [4]. Although SFA containing commodity fats and oils, such as palm and palm stearin, are considered better options in terms of reliability of global supply, pricing as well as their overall physico-chemical attributes, they are perceived as undesirable in terms of potential impact on cholesterol metabolism. All SFAs and SFA sources are not equal in their hypercholesterolaemic effects [5]. Yet, meta-analyses investigating effects of dietary fats on lipid profiles have generally pooled studies that used various dietary sources of SFA [6, 7] potentially resulting in inaccurate conclusions. Cholesterolaemic effects may differ depending on the chemical structure of fatty acids within the dietary SFA [5]. Evidence from animal studies show that whilst long-chain SFA esterified to *sn* 1,3 positions are rapidly cleaved by pancreatic and intestinal lipases and released into the intestine, they are not efficiently absorbed due to the formation of insoluble calcium and magnesium salts and are largely excreted in the faeces [8, 9]. In contrast, the *sn* 2 fatty acid(s) are absorbed efficiently as monoglycerides and transported to the liver where it may influence cholesterol homeostasis differently compared to when occupying *sn*−1,3 positions [5, 10, 11]. Limited evidence exists in humans to support the hypothesis that triacylglycerol (TAG) structure might influence lipid metabolism. Original speculation was based on observations that infants fed breast milk, with palmitic acid (PA) predominantly esterified to the *sn-2* position (~75%) absorbed fat better compared to formulas based on *sn-1,3*-palmitate from palm oil [12]. Although palm olein (PO) is rich in SFA, the *sn-2* position is predominantly occupied by unsaturated fatty acids (~87%, oleic acid and linoleic acid), while in animal fats such as lard, the sn-2 position is mainly SFA (about 87%, PA and stearic acid) [2]. Hence, even though PO and lard have similar proportions of SFA (43 and 39%, respectively) and unsaturated fatty acids (57 and 56%, respectively), they differ in their positional distribution within the TAG molecule. Comparisons between lard and PO on plasma cholesterol outcomes have, however, been inconsistent [13–15]. Similarly, a limited number of small intervention studies that compared effects of increased PA in the *sn*-2 position (using interesterification) to fats with PA in *sn-1,3*, were unable to show differences in cholesterol levels [10, 16, 17]. Nevertheless, more recent animal and human studies using interesterified fats have assigned an important role for fatty acid(s) occupying the sn2 position for several metabolic outcomes [18]

In the present study, we tested the hypothesis that dietary fats high in SFA, but containing mostly unsaturated fatty acids in the *sn-2* position (PO and cocoa butter [CB]) will behave more like an unsaturated fat source (olive oil [OO]) in terms of their effects on lipid profiles, regardless of their total SFA content.

The current study represents the Australian arm of a larger study conducted in several centres, all following the same protocol.

## Subjects/methods

The trial (http://www.anzctr.org.au; ACTRN12616000069459) was conducted at CSIRO’s Nutrition and Health Research Clinic, Adelaide, SA, Australia between May and November 2016 according to National Health and Medical Research Council National Statement on Ethical Conduct in Human Research and ethics approval obtained from CSIRO Human Research Ethics Committee (HREC#15/2015). Participants provided written informed consent.

### Participants

Healthy adults (20–40 years; body mass index (BMI) 18.5– ≤ 27.5 kg/m^2^) were recruited. Exclusion criteria were: abnormal liver and kidney function (elevated alanine transaminase (ALT), aspartate transaminase (AST), creatinine), history of chronic disease, pancreatic insufficiency or fat malabsorption conditions, smoking 6 months prior, lipid/blood pressure (BP) lowering medication, BP > 140/90 mmHg, hyperlipidemia (fasting total cholesterol [TC] > 6.2 mmol/L, TAG > 2.0 mmol/L), allergies to intervention foods, pregnancy/breastfeeding, supplements affecting study outcomes, active weight-loss, hormone-based contraceptives < 3 months prior, hypo-/hyperthyroidism.

### Study design

A 4-week single-blind, randomised 3 × 3 crossover feeding intervention, preceded by 2-week run-in and separated by 2-week washout periods. Participants were stratified by gender and randomly assigned to one of three allocation sequences generated by an individual independent to study allocations using http://www.randomization.com/. Staff involved in data collection and analysis were blind to allocations. Participants and research dietitians were not fully blinded as appearance and flavour of test fats differed.

Outcome measures were assessed pre- and post-intervention. Blood samples were collected following overnight fast; weight and height were measured and BMI calculated; BP were measured using an automated BP monitor [Philips SureSigns VS3]. Participants were asked to maintain usual exercise patterns and limit intake of medications.

### Experimental diets

Participants consumed similar diets differing in test fats only, containing either OO (purchased locally, La Espanola Extra Virgin Olive Oil), PO (IV64) (supplied by Malaysian Palm Oil Board), or CB (Guon Chong Cocoa Manufacturer, Malaysia). Olive oil was chosen as the comparison fat as the fatty acid(s) occupying the sn-2 position of TAGs are comparable to that of the two test fats (PO and CB), namely low SFAs but enriched with monounsaturated fatty acids (MUFAs) (Table 1). Furthermore, the polyphenol content of OO was thought unlikely to affect lipid profiles [19]. During the run-in and washout periods, PO (IV72) was used (Table 1) because of its slightly different composition than the test PO (IV64) and to achieve uniformity across different study centres. As the purpose of the run-in/washout fat is to bring study participants to an equilibrium (all participants consuming the same fat) before changing over to a different intervention fat, the type of fat used was considered irrelevant. Experimental diets delivered 30–35 percentage of total energy (%E) as total fat with 20%E derived from test fats (~44 g fat/day for 8400 kJ diet), ~15%E protein and ~50%E carbohydrate. Test fats were provided via two frozen meals (lunch, dinner), biscuits (consumed daily) and cake (consumed on weekends in lieu of one frozen meal). Nutrient composition of diets was planned using Food Works Professional Edition V7 (Xyris Software, 2012) and re-confirmed analytically using standard AOAC methods as 32%E, 17%E and 44%E from fat, protein and carbohydrate, respectively for all diets. While the diets contained biscuits and cake, the remainder of the diet was made up of healthy foods (fruits, vegetables, wholegrain bread and cereals, low fat dairy products) in order to ensure overall nutritional adequacy (See Supplementary Table 1 for selected daily nutrient intakes from a 8500 kJ diet). In addition, the 2-week run-in period was included to minimise any potential impact that transitioning from habitual diets to intervention diets may have caused.

**Table 1:** Total fatty acid composition and regio-specific distribution of different fatty acids in the triacylglycerols (TAGs) of dietary test fats

% Total FA	Palm olein (IV72)^a^			Palm olein (IV64)			Olive oil			Cocoa butter		
∑SFA	34.5			38.8			19.6			64.5		
∑MUFA	50.7			46.2			65.1			33.1		
∑PUFA	14.8			13.3			15.4			2.5		
TAG Regio-specificity	*sn*-2	*sn*-1,3	*sn-1,2,3*	*sn*-2	*sn*-1,3	*sn-1,2,3*	*sn*-2	*sn*-1,3	*sn-1,2,3*	*sn*-2	*sn*-1,3	*sn-1,2,3*
SFA	4.3	51.3	35.8	8.5	60.7	43.3	nd	28.9	19.5	0	95.9	64.2
MUFA	67.1	41.9	50.2	63.9	34.5	44.3	74.8	60.0	64.8	87.4	4.1	31.7
PUFA	28.6	6.8	14.0	27.6	4.8	12.4	25.2	11.1	15.7	12.6	0	4.1

*MUFA* monounsaturated fatty acid, *nd* not detected, *PUFA* polyunsaturated fatty acid, *SFA* saturated fatty acids. Expressed as % of total fatty acids. Analysed by the Malaysian Palm oil Board (MPOB)

^a^Run-in and washout fat

Meals were prepared in commercial food manufacturing facilities (Community Chef, Victoria, Australia and Kytons Bakery, SA, Australia) according to recipes developed by research dietitian.

Prescribed diets were eucaloric to maintain body weight. Participant’s individual energy requirements were determined using Schofield equation [20]. Diets were designed at 1000 kJ increments and participants assigned the closest 1000 kJ bracket. Remainder of the diet was made up of commercial breakfast cereal (provided), consumed with low fat milk and daily ‘low-fat snacks’ including fruit, low-fat dairy, bread and cereal options (not provided). Snacks could occasionally be swapped for low-fat discretionary product/s from a prescribed list which included alcoholic beverages for flexibility. Participants were requested to consume all foods provided and avoid foods outside of those prescribed.

Compliance to study treatments was monitored by intake checklists and weekly online surveys (Survey Gizmo, Widgix Software, LLC) and cross-monitored by dietitian at each visit. Percentage compliance scores were calculated for each intervention period using data on daily consumption of test fat food items (frozen meals, biscuits and cake) by totalling the reported intake of test fat and dividing it by the prescribed test fat intake for each individual based on their energy level. Subsequently, a mean value was derived for each intervention period. Participants also recorded their body weight (at home); 3 × /week during run-in and 1 × /week during intervention. If trends were observed in body weight changes ( ± ~1 kg during run-in; ± ~3 kg during intervention) or deviation from study protocol, adjustments were made to the prescribed energy intake as required.

### Lipid analysis

Plasma and serum were prepared by centrifugation at 4 °C (2100 g, 10 min; 2850 g, 15 min, respectively). Samples were stored at −80 °C until analysis at completion of intervention. Serum TC, TAG, high density lipoprotein cholesterol (HDL-C), low density lipoprotein cholesterol (LDL-C) and non-esterified fatty acids (NEFA) were analysed using enzymatic kits (Beckman Coulter Inc, CA, USA; Randox Laboratories Ltd, County Antrim, UK) on a Beckman AU480 analyser. Intra-assay coefficient of variances (CVs) were TC: 0.78%; TAG: 0.86%: HDL-C: 0.69%; LDL-C: 1.79%; and NEFA: 1.61%. Serum Lp(a), ApoA1 and ApoB were measured using immunoturbidimetric kits purchased from above-listed suppliers. Intra-assay CVs Lp(a): 0.86%; ApoA1: 0.86%; ApoB: 0.92%. Plasma fatty acids analysed by gas chromatography–mass spectrometry (GCMS) technique [21, 22], stereo-specific analysis of the test fats with nuclear magnetic resonance (NMR) spectroscopy [22] and LDL sub-fractions using Lipoprint testing system (Quantimetrix, USA).

### Statistical analysis

Statistical power for this study is based on *a priori* hypothesis of not detecting a clinically meaningful difference in the primary outcome of TC:HDL-C ratio. A retrospective power calculation indicated that a sample size of 38 (number of completers in the current study) provided sufficient power (80%, two-tailed α = 0.05) to detect a minimum difference of 0.15 mmol/L in TC:HDL-C ratio using standard deviation (SD) difference of 0.32 (calculated from the current sample). A difference of 0.15 mmol/L is associated with < 5% lower ischemic heart disease mortality [23].

Analysis was performed using SPSS software version 23 (IBM Corporation, New York, USA). Outcome variables were examined for normality using Kolmogorov–Smirnov, Shapiro–Wilk tests and normality plots. Non-normally distributed data were transformed into approximate normal distributions by logarithmic transformations (Lp(a), NEFA).

Primary analyses to compare outcome variables within and between test diets were performed using mixed effects longitudinal models. Because of large attrition rates (34%), analyses were performed on completers (*n* = 38) using unstructured repeated covariance matrix structure. Treatment and time were included as fixed factors and analysed for main effects and treatment*time interactions. All analyses were controlled for weight changes. Baseline levels were controlled for when contributing significantly to model. Data were examined for interaction effects due to order in which treatments were consumed by including allocation order as fixed factor in the model and investigating its interaction with treatment*time. This was removed when no allocation order interactions were evident. When significant treatment*time interactions were seen, post-hoc analysis was performed using repeated measures general linear model with Bonferroni adjustments. Results are presented as estimated marginal means (95% CI). Assumption of normally distributed residuals were met.

*P*-values of < 0.05 were considered significant.

## Results

### Participants, diet and compliance

**Fig. 1:**
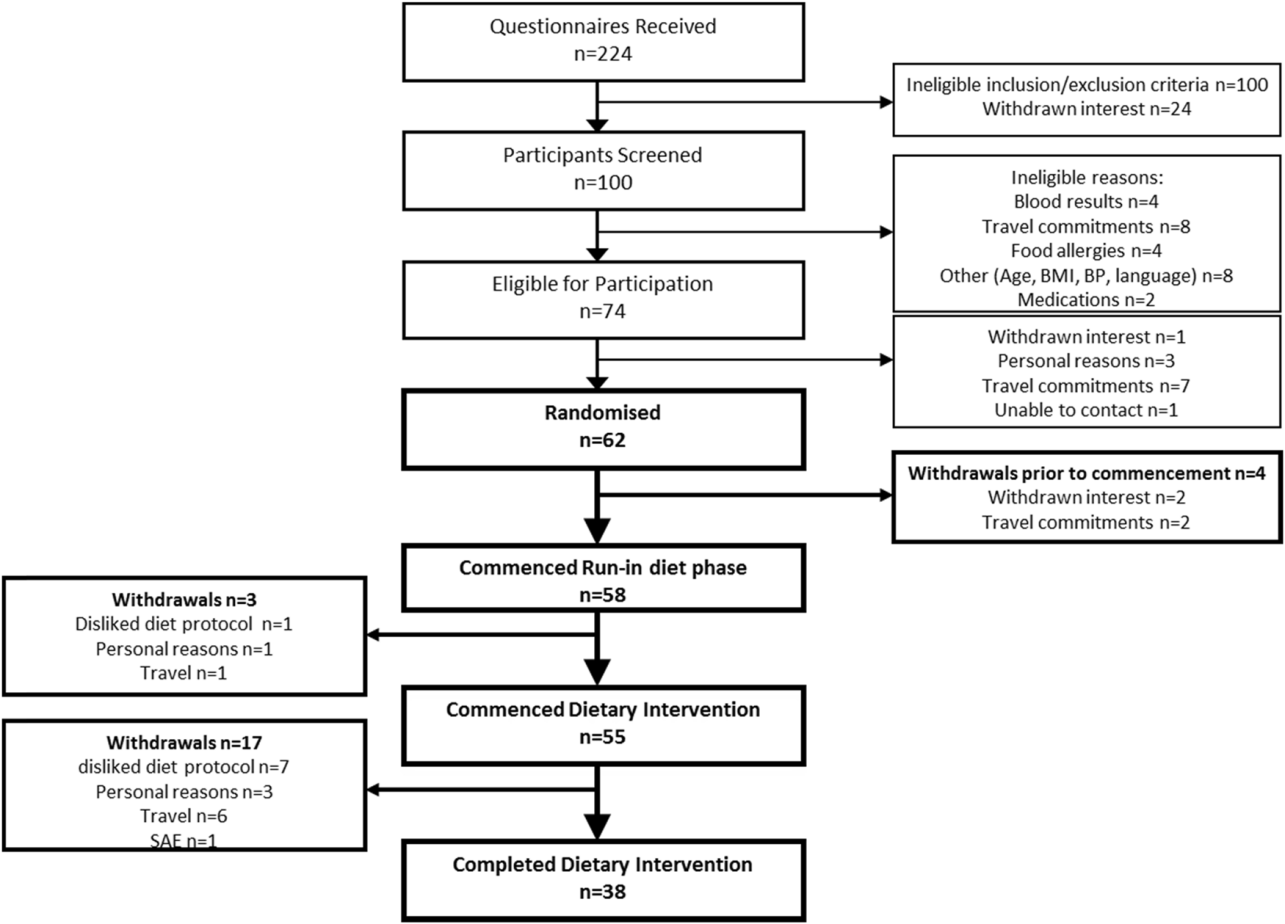
Flow chart of participants through the trial (BMI, Body mass index; BP, Blood pressure; SAE, serious adverse event)

Fifty-eight (58) participants commenced the run-in diet; 38 completed all treatments (Fig. 1). Three participants withdrew during the run-in phase and a further 17 after commencement of intervention. Reasons for withdrawal included dislike of dietary protocol, mostly due to intolerance to meals/snacks (*n* = 7) or restrictions the protocol imposed (*n* = 1), travel commitments (*n* = 7), personal reasons (*n* = 4) and following a serious adverse event (SAE), biliary colic (*n* = 1).

Mean compliance for completers were CB 97%, OO 99%, PO IV64 98% and palm olein IV72 (control) 99%. Dietary protocol deviations were small; 83 deviations (~2.3/participant) recorded for the consumption of other high-fat foods over the 18 weeks.

Baseline characteristics (*n* = 55) (Table 2) reflect inclusion criteria; namely young healthy adults with anthropometric, blood pressure and serum lipid profiles within normal ranges. Baseline characteristics did not differ between completers and withdrawals (*P* > 0.05) except for serum TC that was slightly higher in withdrawals compared to completers (mean [SD], 4.79 [0.65] mmol/L versus 4.33 [0.83] mmol/L, *P* = 0.05).

**Table 2:** Baseline Characteristics (*n* = 55)^a^

Variable	Mean (SD)
Age (years)	29.8 (4.77)
Weight (kg)	68.9 (11.3)
Height (m)	1.74 (0.10)
BMI (kg/m^2^)	23.0 (2.18)
Waist circumference (cm)	74.0 (7.04)
Systolic blood pressure (mmHg)	109 (8.30)
Diastolic blood pressure (mmHg)	68.5 (7.04)
Mean arterial pressure (mmHg)	81.7 (6.90)
Heart rate (beats/minute)	68.4 (10.8)
Serum TC (mmol/L)	4.47 (0.80)
Serum TC/HDL-C ratio (mmol/L)	3.14 (0.69)
Serum TAG (mmol/L)	0.87 (0.31)
Serum LDL-C (mmol/L)	2.77 (0.60)
Serum HDL-C (mmol/L)	1.46 (0.33)

*BMI* body mass index, *HDL-C* high-density lipoprotein cholesterol, *LDL-C* low-density lipoprotein cholesterol, *TAG* triacylglycerol, *TC* total cholesterol

^a^All participants who commenced the dietary interventions

Allocation order affected body weight and BMI (time*treatment*allocation order, *P* < 0.01) and is explained by a significant reduction in body weight and BMI during the 1st phase of the trial in the group who consumed OO first compared to CB and PO. No further changes were seen in weight or BMI during the trial with any of the dietary fats (Supplementary Table 2). Consequently, all statistical analyses were controlled for changes in body weight.

Changes in BP were not different between test diets (Supplementary Table 2).

Plasma PA (C16:0) levels decreased with the intake of OO versus PO. Plasma stearic acid (C18:0) increased with CB compared to PO. Plasma oleic acid (OA; C18:1) and total MUFA levels increased with OO compared to PO and CB (Table 3).

**Table 3:** Changes in selected plasma fatty acid levels (% of total fatty acids) from baseline and comparisons between treatments (*n* = 38)

Variable	Time (wk)	CB	OO	PO	*P*-values^a^
Myristic acid (C14:0)	0	0.61 (0.52, 0.71)	0.53 (0.46, 0.60)	0.52 (0.46, 0.57)	–
	Δ4	−0.04 (−0.12, 0.05)	0.01 (−0.08, 0.11)	0.06 (−0.001, 0.13)	0.28
Palmitic acid (C16:0)	0	25.6 (24.2, 27.0)	27.6 (25.8, 29.4)	25.6 (24.4, 26.9)	–
	Δ4	−1.96 (−3.77, −0.15)^b^	−3.53 (−5.92, −1.15)^b,c^	0.65 (−1.02, 2.32)	0.01
Stearic acid (C18:0)	0	8.79 (8.04, 9.55)	9.20 (8.27, 10.1)	9.28 (8.62, 9.93)	–
	Δ4	1.35 (0.32, 2.39)^b,d^	−0.78 (−1.90, 0.33)	−0.48 (−1.13, 0.18)	0.01
Oleic acid (C18:1n-9cis)	0	18.0 (16.2, 19.7)	15.9 (13.7, 18.1)	17.2 (15.5, 18.9)	–
	Δ4	2.44 (0.41, 4.48)^b^	5.10 (2.90, 7.30)^b,c^	0.63 (−0.68, 1.95)	0.002
Linoleic acid (C18:2n-6cis)	0	24.7 (23.1, 26.2)	23.2 (20.9, 25.6)	25.0 (23.6, 26.5)	–
	Δ4	−0.73 (−2.82, 1.35)	0.25 (−2.35, 2.84)	−0.09 (−1.90, 1.72)	0.80
Arachidonic acid (C20:4n-6)	0	5.39 (4.37, 6.42)	4.82 (3.78, 5.85)	5.59 (4.63, 6.56)	–
	Δ4	0.77 (−0.08, 1.61)	1.08 (0.30, 1.87)	0.19 (−0.47, 0.84)	0.18
Total SFA	0	36.5 (32.6, 40.5)	37.1 (31.3, 42.9)	37.8 (35.6, 39.9)	–
	Δ4	−1.30 (−4.21, 1.62)	−0.06 (−5.77, 5.65)	−1.28 (−3.42, 0.85)	0.94
Total MUFA	0	25.3 (24.1, 26.4)	24.6 (23.4, 25.7)	24.4 (23.4, 25.4)	–
	Δ4	0.79 (−0.49, 2.08)	4.05 (2.57, 5.53)^b,c,e^	0.18 (−0.67, 1.03)	<0.001
Total PUFA	0	36.7 (35.1, 38.2)	34.7 (32.5, 36.9)	36.8 (35.3, 38.3)	–
	Δ4	−0.51 (−2.87, 1.86)	0.33 (−2.12, 2.78)	−0.84 (−2.80, 1.12)	0.79

*CB* cocoa butter, *MUFA* monounsaturated fatty acids, *OO* olive oil, *PO* palm olein, *PUFA* polyunsaturated fatty acids, *SFA* saturated fatty acids, *Δ* estimated marginal mean (95%CI)

^a,b^Comparisons within and between treatment groups were performed using mixed effects longitudinal models

^b^Change significantly different from baseline (*P* < 0.05)

^c,d,e^Post-hoc comparisons between treatments significantly different (*P* < 0.05) (^c^, OO vs. PO; ^d^, CB vs. PO; ^e^, OO vs. CB); analysis performed using Repeated measures General Linear Model with Bonferroni adjustments for multiple comparisons

No differences were seen between any diets on serum lipids, including TC:HDL-C, TC, LDL-C, TAG, HDL-C, ApoA1, ApoB, Lp(a) or NEFA (Table 4). Significant allocation order interactions occurred for TC, LDL-C and ApoB, but the overall result of no difference between diets did not change. Those who consumed the diets in the order PO-OO-CB showed increases in LDL-C during the PO diet compared to those who received the diets in the order OO-CB-PO. A trend was seen for LDL-C to reduce during the OO diet compared to PO diet (*P* = 0.05) and for serum apoA1 to decrease with CB diet (*P* = 0.05).

**Table 4:** Changes from baseline in serum lipid profiles and comparisons between treatments (*n* = 38)

Variable	Time (wk)	CB	OO	PO	*P*-values^a^
TC (mmol/L)	0	4.48 (4.22, 4.75)	4.40 (4.14, 4.66)	4.48 (4.21, 4.73)	–
	Δ4	−0.05 (−0.18, 0.08)	−0.04 (−0.20, 0.11)	0.06 (−0.04, 0.16)	0.14
LDL-C (mmol/L)	0	2.78 (2.60, 2.97)	2.73 (2.53, 2.93)	2.79 (2.59, 2.99)	–
	Δ4	0.00 (−0.12, 0.12)	−0.05 (−0.17, 0.08)	0.07 (−0.01, 0.14)	0.05
HDL-C (mmol/L)	0	1.47 (1.35, 1.59)	1.44 (1.33, 1.55)	1.46 (1.36, 1.56)	–
	Δ4	−0.05 (−0.11, 0.0)	0.0 (−0.04, 0.04)	−0.01 (−0.04, 0.02)	0.31
TC/HDL-C (mmol/L)	0	3.15 (2.95, 3.35)	3.14 (2.92, 3.37)	3.14 (2.93, 3.36)	–
	Δ4	0.06 (−0.05, 0.16)	−0.01 (−0.15, 0.13)	0.08 (0.00, 0.15)	0.53
TAG (mmol/L)	0	0.91 (0.80, 1.03)	0.85 (0.75, 0.96)	0.82 (0.73, 0.92)	–
	Δ4	−0.07 (−0.15, 0.01)	0.01 (−0.09, 0.10)	0.03 (−0.03, 0.08)	0.07
ApoA1 (g/L)	0	1.49 (1.40, 1.59)	1.46 (1.37, 1.55)	1.47 (1.39, 1.55)	–
	Δ4	−0.07 (−0.11, −0.02)^b^	0.01 (−0.02, 0.05)	0.00 (−0.03, 0.04)	0.05
ApoB(g/L)^c^	0	0.79 (0.74, 0.83)	0.79 (0.73, 0.84)	0.79 (0.74, 0.85)	–
	Δ4	0.00 (−0.02, 0.03)	−0.02 (−0.05, 0.02)	0.01 (−0.01, 0.03)	0.18
ApoA1:ApoB	0	1.95 (1.80, 2.09)	1.91 (1.78, 2.04)	1.91 (1.77, 2.05)	–
	Δ4	−0.09 (−0.18, −0.01)^b^	0.03 (−0.05, 0.10)	−0.03 (−0.07, 0.02)	0.13
Lp(a) (nmol/L)^c^	0	33.7 (20.7, 46.7)	36.4 (21.8, 51.1)	32.6 (20.4, 44.9)	–
	Δ4	1.85 (−1.42, 5.12)	0.34 (−1.66, 2.34)	1.96 (−1.94, 5.86)	0.42
NEFA (mmol/L)^c^	0	0.39 (0.29, 0.50)	0.36 (0.28, 0.44)	0.42 (0.31, 0.54)	–
	Δ4	−0.06 (−0.17, 0.06)	0.21 (−0.06, 0.10)	−0.02 (−0.12, 0.08)	0.79

All values are estimated marginal means (95%CI)

*HDL-C* high-density lipoprotein cholesterol, *LDL-C* low-density lipoprotein cholesterol, *Lp(a*) lipoprotein (a), *NEFA* non-esterified fatty acids, *TAG* triacylglycerol, *TC* total cholesterol

^a,b^Comparisons within and between treatment groups were performed using mixed effects longitudinal models. All analyses were controlled for weight changes during the study. Models for TC, LDL-C and ApoB in addition included the following variables that significantly contributed to the model: time*treatment*allocation order and baseline levels

^b^Change significantly different from baseline (*P* < 0.05)

^c^Analyses were performed on log transformed data

LDL sub-fractions detected were mostly large particles, LDL-1 and LDL-2, with no LDL sub-fractions 4–7 detected (Table 5). No differences were seen between the different diets except for serum IDL-A which decreased with OO versus PO (estimated marginal mean (95%) differences in changes: −2.26 (−4.31, −0.21) mg/dL; −1.17 (−2.31, −0.03)%, Bonferroni adjusted *P* < 0.05).

**Table 5:** Changes from baseline in serum lipoprotein sub-fractions and comparisons between treatments (*n* = 38)

Variable	Time (wk)	CB	OO	PO	*P*-values^a^
VLDL (mg/dL)	0	23.1 (21.1, 25.1)	21.5 (20.0, 23.1)	21.6 (19.8, 23.5)	–
	Δ4	−1.17 (−2.91, 0.58)	0.12 (−1.31, 1.54)	0.05 (−1.08, 1.18)	0.46
IDL-C (mg/dL)	0	18.5 (17.0, 20.1)	17.6 (15.9, 19.4)	18.8 (17.4, 20.3)	–
	Δ4	−0.87 (−1.74, 0.00)	−0.85 (−2.30, 0.60)	−0.13 (−1.2, 0.93)	0.52
IDL-B (mg/dL)	0	9.12 (8.32, 9.93)	10.0 (7.76, 12.3)	9.27 (8.17, 10.4)	–
	Δ4	−0.07 (−0.71, 0.57)	−1.35 (−3.29, 0.58)	1.27 (−0.83, 3.38)	0.17
IDL-A (mg/dL)	0	18.1 (15.9, 20.4)	18.3 (15.9, 20.7)	18.3 (15.8, 20.8)	–
	Δ4	0.55 (−0.92, 2.01)	−1.80 (−3.41, −0.18)^b,c^	0.47 (−0.73, 1.66)	0.03
LDL-1 (mg/dL)	0	35.0 (31.8, 38.2)	34.0 (30.8, 37.2)	34.8 (32.1, 37.5)	–
	Δ4	1.67 (−0.18, 3.52)	−0.07 (−2.62, 2.47)	0.59 (−1.58, 2.76)	0.5
LDL-2 (mg/dL)	0	11.4 (8.57, 14.2)	11.3 (8.13, 14.5)	11.3 (8.05, 14.5)	–
	Δ4	0.28 (−1.07, 1.64)	1.52 (−0.58, 3.62)	0.19 (−1.43, 1.80)	0.41
LDL-3 (mg/dL)	0	0.50 (0.15, 0.85)	0.72 (−0.05, 1.50)	0.74 (0.02, 1.46)	–
	Δ4	−0.07 (−0.40, 0.27)	0.04 (−0.80, 0.87)	−0.38 (−1.00, 0.22)	0.09
VLDL (%)	0	13.4 (12.4, 14.4)	12.9 (12.1, 13.7)	12.6 (11.7, 13.5)	–
	Δ4	−0.50 (−1.27, 0.26)	0.23 (−0.41, 0.86)	−0.11 (−0.68, 0.46)	0.38
IDL-C (%)	0	10.7 (10.1, 11.3)	10.5 (9.64, 11.3)	11.0 (10.4, 11.6)	–
	Δ4	−0.43 (−0.82, −0.04)	−0.31 (−0.89, 0.26)	−0.26 (−0.86, 0.34)	0.88
IDL-B (%)	0	5.25 (4.86, 5.64)	5.95 (4.73, 7.17)	5.29 (4.89, 5.69)	–
	Δ4	−0.03 (−0.39, 0.33)	−0.73 (−1.80, 0.34)	0.81 (−0.42, 2.03)	0.17
IDL-A (%)	0	10.5 (9.32, 11.7)	11.0 (9.69, 12.3)	10.6 (9.30, 11.9)	–
	Δ4	0.44 (−0.32, 1.19)	−1.00 (−1.89, −0.12)^b,c^	0.23 (−0.41, 0.87)	0.03
LDL-1 (%)	0	20.1 (19.2, 21.0)	19.7 (18.3, 21.1)	20.1 (19.0, 21.2)	–
	Δ4	1.01 (0.11, 1.92)^b^	0.52 (−0.94, 1.98)	0.07 (−1.16, 1.29)	0.45
LDL-2 (%)	0	6.47 (4.96, 7.97)	6.37 (4.82, 7.92)	6.34 (4.73, 7.94)	–
	Δ4	0.14 (−0.59, 0.87)	1.17 (0.12, 2.22)^b^	0.07 (−0.71, 0.85)	0.13
LDL-3 (%)	0	0.27 (0.09, 0.45)	0.37 (0.05, 0.70)	0.39 (0.08, 0.70)	–
	Δ4	−0.03 (−0.20, 0.14)	0.07 (−0.29, 0.43)	−0.19 (−0.43, 0.05)	0.09

All values are estimated marginal means (95%CI)

*CB* cocoa butter, *IDL* intermediate-density lipoprotein, *LDL-C* low-density lipoprotein, *OO* olive oil, *PO* palm olein, *VLDL* very low-density lipoprotein, cholesterol

^a,b^Comparisons within and between treatment groups were performed using mixed effects longitudinal models. All analyses were controlled for weight changes during the study. Models for IDL-C%, IDL-C mg/dL, IDA-A mg/dL and LDL-1 mg/dL also included time*treatment*allocation order

^b^Change significantly different from baseline (*P* < 0.05)

^c^Post-hoc comparisons between OO vs. PO treatments significantly different (*P* < 0.05); analysis performed using Repeated measures General Linear Model with Bonferroni adjustments for multiple comparisons

Including all participants, a total of 78 AEs (adverse events) were reported. None were associated with specific fat types. Most AEs were mild to moderate and related to respiratory infections, hence likely seasonal. Five AEs related to gastrointestinal/intolerance were considered possibly related to study diets including one SAE, biliary colic. One female participant was diagnosed with low iron status, possibly related to the study diets as she was on the lowest energy intake bracket providing less iron (12.8 mg/day) than the RDI (18 mg/day).

## Discussion

The present study was designed to evaluate the assumption that the sn-2 position of dietary TAGs is a key determinant of plasma lipid outcomes of dietary fats and oils. As hypothesised, no differences were seen between test fats on the primary outcome, TC:HDL-C ratio, as well as a range of other lipid variables (TC, LDL-C, HDL-C, TAG, apoA1, apoB, apoA1:apoB, Lp(a), NEFA) and LDL sub-fractions.

Consistent with the current investigation, other studies in Australia and Malaysia using similar trial designs considering duration, amount of fat and study population, showed comparable non-hypercholesterolaemic effects between PO and OO [24–26]. A study in Danish men reported neutral effects of OO and PO on the TC:HDL-C ratio, but the OO diet resulted in a small (4.5%) significant decrease in plasma TC and LDL-C levels compared to the PO diet [14]. A recent meta-analysis also showed no effect of PO compared to MUFA-rich fats (6 studies) or PO compared to stearic acid (3 studies) on TC:HDL-C ratio [27]. Although a trend was seen for more favourable changes in serum LDL-C after the OO diet compared to the PO diet, the mean differences in LDL-C were relatively small (0.12 mmol/L); associated with an estimated coronary heart disease (CHD) mortality risk reduction of only ~3% [28].

Only a few dietary trials used CB sources [29, 30] while several used shea butter as the source of stearic acid [31–33], the main fatty acid in CB, generally demonstrating cholesterol-neutral properties. Shea butter has a similar stereospecificity compared to CB with most SFA in *sn*−1,3 position [32], but are lower in PA (4% versus 26%) and higher in oleic acid (OA) (49% versus 33%) and linoleic acid (7% versus 2.5%) compared to CB [34]. The non-hypercholesterolaemic effects of stearic acid may be explained by enhanced faecal excretion [31] due to its stereospecificity [35] and by rapid conversion of stearic acid to OA [33, 36]. In support of the latter, the current study showed a significant increase from baseline to end in plasma OA with the CB diet.

Assessment of LDL particle size provides additional information regarding the atherogenetic potential of dietary interventions. Participants in the current study had mostly large, buoyant, less atherogenic [37, 38] LDL particles (serum LDL-1 and -2 sub-fractions) and although the diets did not differ with regard to their effects on LDL sub-fractions; it is important to note that none of the diets induced lipoprotein sub-fraction populations/profiles deemed more atherogenic. The only notable change in lipoprotein sub-fractions occurred following the OO diet where reduced levels of serum IDL-A compared to PO was observed. IDL’s are triglyceride-rich particles as they originate from the catabolism of very low-density lipoprotein (VLDL) by lipoprotein lipase [39]. Increased IDL have been reported to promote the progression of atherosclerosis [40, 41]. Whilst a difference was registered in IDL-A between the OO and PO diets, this change was due to a decrease in response to the OO diet while no change was observed after the PO diet. The exact origin of this lipoprotein sub-fraction is not clearly evident from the present findings as none of the major lipid parameters (LDL-C, HDL-C and triglycerides) were perturbed by any of the diets.

Although the current study hypothesis was based on plasma lipid outcomes in response to the type of fatty acid(s) esterified to the *sn*-2 position of TAGs, the present design was not optimal for direct testing of this theory. A better approach would have been to directly compare the same fat source with fatty acids distribution in the *sn*-2 position versus *sn*-1,3 positions, for example, structured TAGs where PA is esterified to *sn-2* position (*sn-2* palmitate). Hence, the non-hypercholesterolaemic effects of the two test fats rich in SFA compared to OO could have been due to a combination of reasons including the *sn*-2 position itself, the presence of relatively high proportion of OA (>30% in CB and ~45% PO), presence of tocotrienols and plant sterols in PO [42] or the rapid conversion of stearic acid to OA in the case of CB [33, 36].

A strength of the current study is the inclusion of a range of traditional and new emerging lipid markers of CVD risk. Most previous studies focused on TC and LDL-C, providing limited view of the atherogenic potential of a given fat type. The primary outcome, TC:HDL-C ratio, provides a better overall indication of CHD risk and has been shown to be a stronger predictor of ischaemic heart disease mortality than TC alone. Serum apoB is considered an important emerging CVD risk stratification marker and has been shown to perform better than LDL-C in CVD risk prediction [43]. Recent results from the prospective PURE study, involving 18 countries, *n* = 125,287, suggested apoB/apoA1 ratio was the best overall indicator of the effect of SFA on CVD risk [44]. Results from the current study consistently showed no difference between dietary fat types on any of these lipid markers, increasing overall confidence in the findings.

Other strengths include rigorous dietary intake control; a highly controlled feeding protocol, where the test fats were delivered in meals and snacks to participants for the duration of the study 7 days a week. Reported compliance was high (98% + ) which was confirmed by changes in plasma fatty acids that mirrored composition of the source fats. The high intake of test fats (20%E, two-thirds of total fat intake) ensured that if an effect was present it would have been apparent. PO and CB are not common in the typical Australian diet and are most likely only consumed through commercial food products like bakery and fried products, hence intakes of these fat types are typically much lower than the amounts consumed in this study. Furthermore, the study had sufficient statistical power to detect clinical meaningful differences in the primary outcome variable and most lipid variables.

The allocation order interactions seen for TC, LDL-C and ApoB is a limitation. Those who consumed the diets in the order PO-OO-CB showed significant increases in LDL-C during the PO diet compared to those who received the diets in the order OO-CB-PO. This may have been instigated by a slightly lower LDL-C levels at baseline in the PO-OO-CB group (2.66 [2.30, 3.02] mmol/L) compared to the OO-CB-PO group (2.84 [2.54, 3.15] mmol/L). The reduction in body weight and BMI in the group consuming OO in the first phase of the trial may have further aggravated this effect, although weight changes were controlled for in the statistical analysis. Despite these allocation order interactions, the overall result of no difference between diets did not change.

Other potential limitations of the study include that results can only be generalised to healthy young adults; hence it is unclear how these fat types may affect obese, hypercholesterolaemic individuals, children or older adults.

In conclusion, consistent with the hypothesis, PO and CB both rich in SFA, but containing primarily unsaturated fatty acid in the *sn-2* position of TAG, did not differ from OO with regard to their effects on lipid profiles.

## Supplementary information

Supplementary Table 1

Supplementary Table 2
