# Supplementary material for: Fatty acid regio-specificity of triacylglycerol molecules may affect plasma lipid responses to dietary fats—a randomised controlled cross-over trial
Source: Eur J Clin Nutr. 2019 Jun 21;74(2):268–77. doi: 10.1038/s41430-019-0452-7 (PMC7007409; doi:10.1038/s41430-019-0452-7)
Supplement: Supplementary file 1 — Supplementary Table 1 [file 41430_2019_452_MOESM1_ESM.docx]

**Supplementary Table 1. Selected daily nutrient intakes from 8500kJ diet^1^**

| **Nutrient** | **Daily Amount** |
| --- | --- |
| Energy (kJ) | 8829 |
| Protein (g) | 95.6 |
| Protein (% Energy) | 18.4 |
| Total fat (g) | 74.9 |
| Total fat (% Energy) | 31.4 |
| Carbohydrate (g) | 249.2 |
| Carbohydrage (% Energy) | 46.5 |
| Sugar (g) | 110.2 |
| Starch (g) | 137.8 |
| Dietary fibre (g) | 26.0 |
| Thiamin (mg) | 1.6 |
| Riboflavin (mg) | 2.2 |
| Niacin (mg) | 21.5 |
| Niacin equivalents (mg) | 39.0 |
| Vitamin C (mg) | 107.6 |
| Vitamin E (mg) | 15.4 |
| Alpha tocopherol (mg) | 13.3 |
| Vitamin B6 (mg) | 1.6 |
| Vitamin B12 (µg) | 5.6 |
| Folate (µg) | 412.8 |
| Vitamin A equivalents (µg) | 954.8 |
| Retinol (µg) | 66.0 |
| Beta carotene equivalents (µg) | 5340.7 |
| Beta carotene (µg) | 3832.7 |
| Sodium (mg) | 1792.0 |
| Potassium (mg) | 3364.4 |
| Magnesium (mg) | 302.3 |
| Calcium (mg) | 910.5 |
| Phosphorus (mg) | 1559.4 |
| Iron (mg) | 12.7 |
| Zinc (mg) | 43.8 |

^1^ Nutritional analysis performed in Food Works Professional Edition version 7 (Xyris Software, 2012)
