# Supplementary material for: Fatty acid regio-specificity of triacylglycerol molecules may affect plasma lipid responses to dietary fats—a randomised controlled cross-over trial
Source: Eur J Clin Nutr. 2019 Jun 21;74(2):268–77. doi: 10.1038/s41430-019-0452-7 (PMC7007409; doi:10.1038/s41430-019-0452-7)
Supplement: Supplementary file 2 — Supplementary Table 2 [file 41430_2019_452_MOESM2_ESM.docx]

**SUPPLEMENTARY** **TABLES**

Supplementary Table 2 Changes from baseline in anthropometric measurements and blood pressure and comparisons between treatments (n=38)

| **Variable** | **Time (wk)** | **CB** | **OO** | **PO** | **P-values^a^** | **Estimated marginal mean (95%) differences in changes between groups^c^** | | |
| --- | --- | --- | --- | --- | --- | --- | --- | --- |
|  |  |  |  |  |  | **CB *vs*. OO** | **CB *vs*. PO** | **OO *vs*. PO** |
| Weight (kg) | 0 | 68.2 (64.6, 71.8) | 68.3 (64.6, 72.0) | 68.6 (64.9, 72.3) | - | - | - | - |
|  | Δ4 | 0.07 (-0.28, 0.41) | -0.41 (-0.69, -0.12)^b^ | -0.05 (-0.31, 0.22) | 0.03 | 0.48 (-0.05, 1.00) | 0.11 (-0.38, 0.61) | -0.36 (-0.74, 0.02) |
| Waist (cm) | 0 | 74.5 (72.2, 76.8) | 74.4 (72.0, 76.8) | 74.4 (72.0, 76.8) | - | - | - | - |
|  | Δ4 | -0.32 (-0.94, 0.31) | -0.02 (-0.68, 0.64) | 0.32 (-0.27, 0.90) | 0.22 | - | - | - |
| BMI (kg/m^2^) | 0 | 22.7 (22.0, 23.4) | 22.7 (22.0, 23.5) | 22.8 (22.1, 23.5) | - | - | - | - |
|  | Δ4 | 0.01 (-0.10, 0.12) | -0.14 (-0.23, -0.04)^b^ | -0.02 (-0.11, 0.06) | 0.04 | 0.15 (-0.02, 0.31) | 0.03 (-0.13, 0.19) | -0.12 (-0.24, 0.01) |
| SBP (mmHg) | 0 | 109 (107, 110) | 111 (109, 114) | 109 (107, 112) | - | - | - | - |
|  | Δ4 | 0.44 (-1.76, 2.65) | -2.30 (-4.43, -0.16)^b^ | 1.44 (-1.11, 3.98) | 0.09 | - | - | - |
| DBP (mmHg) | 0 | 67.6 (65.4, 69.9) | 69.4 (67.3, 71.6) | 67.8 (65.7, 69.9) | - | - | - | - |
|  | Δ4 | 0.33 (-1.73, 2.38) | -1.07 (-3.24, 1.11) | 0.52 (-1.39, 2.43) | 0.56 | - | - | - |

BMI, body mass index; CB, cocoa butter; DBP, diastolic blood pressure; OO, olive oil; PO, palm olein; SBP, systolic blood pressure
Δ, estimated marginal mean (95%CI)
^a,b^Comparisons within and between treatment groups were performed using mixed effects longitudinal models. For weight, waist and BMI changes analyses, time*treatment*allocation order was added as it significantly contributed to the model. For blood pressure changes analyses weight changes during the study and baseline levels were controlled for
^b^Change significantly different from baseline (*P*<0.05)
^c^Post-hoc comparisons between groups were performed using Repeated measures General Linear Model. However, none of the differences between groups were significant (*P*>0.05) after Bonferroni adjustments for multiple comparisons were made.
